# Influence of methadone on the anticonvulsant efficacy of valproate sodium gabapentin against maximal electroshock seizure in mice by regulation of brain MDA TNF-α

**DOI:** 10.3389/fneur.2022.920107

**Published:** 2022-08-23

**Authors:** Ali Moradi Jafari, Majid Hassanpourezatti

**Affiliations:** Department of Biology, Basic Sciences School, Shahed University, Tehran, Iran

**Keywords:** methadone, valproate sodium, gabapentin, maximal electroshock seizures, malondialdehyde, tumor necrosis factor-alpha, mice

## Abstract

Methadone is the most frequently used opioid therapy worldwide, with controversial effects on oxidative stress homeostasis. This study investigated the effects of intraperitoneal (i.p.) co-administration of methadone (0.1, 0.3, 1, and 3 mg/kg) and valproate sodium (300 mg/kg) or gabapentin (50 mg/kg) in the mice maximal electroshock (MES)-induced seizure model. The adverse effect of drugs was assessed using the chimney test. The levels of tumor necrosis factor-alpha (TNF-α) and malondialdehyde (MDA) contents were measured in mice brains after a single seizure. Administration of methadone alone resulted in a significant reduction in the duration of hind limb extension (HLE) than that in the control group. Methadone pretreatment at doses of 0.1 and 0.3 mg/kg i.p. decreased, and at doses of 1 and 3 mg/kg i.p. had an increasing effect on anticonvulsant efficacy of gabapentin. Pretreatment with all doses of methadone significantly decreased the valproate anticonvulsive efficacy. At doses of 1 and 3 mg/kg i.p. methadone *per se* increased brain MDA levels after MES-induced seizure. Administration of methadone (0.3 mg/kg i.p.) enhanced and at 3 mg/kg decreased gabapentin effect on brain MDA level, but their co-treatment did not lead to further increase in MDA. Methadone at 0.3–3 mg/kg enhanced the effect of sodium valproate on MDA levels in the brain, but at all doses significantly potentiated its effect on brain TNF-α levels. The drugs did not produce any side effects on motor coordination in experimental animals. In conclusion, methadone showed different effects on anticonvulsant actions of gabapentin and valproate through regulation of brain levels of MDA and TNF-α.

## Introduction

Despite acceptable progress, complete seizure control is not achieved in >30% of patients with epilepsy (1). Polytherapy is a promising approach recently proposed to treat resistant epilepsy and the actual prevention of the disease (2). Animal models were used to preselect suitable combinations of drugs with anticonvulsants that could finally be evaluated in clinics. Targeting endogenous opioid systems due to their anticonvulsant and neuroprotective properties has been proposed as a new therapeutic approach for epilepsy treatment (3). The literature review indicated that opioid drugs, either directly or in combination with other anticonvulsant medications, might lead to contradictory results in terms of seizure control (4–6). It was suggested that the accumulation of cytokines and lipid peroxidation products in the brain might lead to abnormal and excessive simultaneous firing in a group of neurons and epilepsy (7). Therefore, exploring the role of lipid peroxidation and cytokines in the regulation epilepsy-related processes may help us understand the molecular mechanisms behind epilepsy and also predict the success of this combination therapy in the treatment of epilepsy (8).

Previously, the combination of opioids and gabapentin prescription was used to treat pain beyond the usual medical condition treatment, and valproate was used as an analgesic adjuvant to reduce the development of morphine side effects (9, 10). The opioid agonists have been recognized for having potential for both proconvulsant and anticonvulsant effects in experimental models of epilepsy (11–14). Methadone is a synthetic mu receptors agonist used for opioid maintenance therapy. Furthermore, it has several pharmacological activities in addition to opioid activity, such as the antitumor, anti-inflammatory, and antioxidant activities (15, 16). This compound, as a neuromodulator, can suppress intrinsic nerve excitability (17, 18). It has also shown inhibitory effects on both T- and L-type calcium channels in neuroblastoma cells (19, 20). In addition, prenatal exposure of neonate rats with methadone caused a reduction in the levels of GABA proteins in the brain, reversible inhibitory effect on NMDA receptors (21), and acts as a regulator of excitation/inhibition balance in neuronal networks (22–25). GABA proteins are proteins that are directly connected through the intracellular domain to GABA-A receptors and regulate the membrane traffic of these receptors (26).

It prolonged the inactivation period of the neuronal sodium channel and suppressed the action potential firing in peripheral nerves (26, 27). It can be expected that the drug will also be able to influence the anticonvulsant activity of these drugs. So, studying the effect of methadone in combination with gabapentin or valproate in experimental model could significantly increase our knowledge about the mechanisms underlying seizure control in the brain.

Although the pathogenesis of epilepsy has not been fully clarified, the activation of neuroinflammatory and oxidative stress is an important perpetuating factor for epileptogenesis in drug-resistant patients (28). Tumor necrosis factor-alpha (TNF-α), one of the inflammatory markers, is produced and released from epileptic neurons (29). It induces a rapid and persistent decrease in inhibitory synaptic strength and downregulates GABA receptors in mature mouse hippocampal neurons (30). Several lines of evidence suggest that TNF-α potentiates excitatory transmission in both physiological and pathological conditions (31). Indeed, TNF-α plays an essential role in the maximal electroshock (MES)-induced seizure model (32). It was shown that lipid peroxidation products, together with TNF-α, lead to the development of epilepsy by induction of irreversible damage to phospholipids in neuronal cells (33). It has been shown that malondialdehyde (MDA), a lipid peroxidation product, can modify neuronal excitability by regulating the balance between excitatory and inhibitory neurotransmitters in the brain (34). Thus, there is a need for more in-depth knowledge about oxidative stress and inflammatory factors in the pathogenesis of seizures. Further analysis of these factors may better address the effects of anticonvulsant drugs on preventing seizure.

Accordingly, we hypothesized that methadone administration might improve the response to anticonvulsant therapy. We also examine the possible role of brain MDA and TNF-α in these interactions. The effects of treatments on motor coordination were also investigated using the chimney test.

## Materials and methods

### Experimental procedures

#### Drugs

Methadone hydrochloride was purchased from TEMAD Company, Tehran, Iran. Sodium valproate and methadone were received as gifts from Raha Daru and Meher Daru pharmaceutical companies, Tehran, Iran, respectively. Drugs were dissolved in phosphate-buffered saline (PBS) and serially diluted to require doses. Methadone, sodium valproate, and gabapentin were injected intraperitoneally (i.p.) 30, 45, and 60 min, respectively, before the MES-induced seizures based on previous data in the literature (35–37).

#### Animals

Adult male NMRI mice, procured from the Animal House of Shahed University, were used. The animals were housed four per cage in standard cages under environmental conditions with temperature: 22 ± 1°C; humidity: 60 ± 5%; reversed 12-h light/dark cycle with lights turned on at 19:00, and free access to food palate and water *ad libitum*. Mice were only used once in each experiment, and a new group of mice was used for each dose and drug tested. The experiments were performed from 08:00 to 14:00. All experiments were performed in accordance with the NIH Guide for the Care and Use of Laboratory Animals (NIH, 1996). The research protocol was approved by the local ethical committee of Shahed University with the code number IR.SHAHED.REC.1400.072. An effort was made to reduce animal numbers and suffering.

### Behavioral evaluations

#### MES-induced seizure model

Electroconvulsions were generated by alternating current (10 Hz, 37.2 mA, and 0.2 s) delivered *via* ear-clip electrodes connected to a stimulator apparatus (Borj Sanat Co. Tehran-Iran) as described by Jahani et al. (38). Electrodes were moistened with saline solution before each application, and the mice were observed in the duration of hind limb extension (HLE) in mice. The reduction in HLE duration was an index of anticonvulsant success. The percentages of protection were calculated according to the following formula:

Protection % = 100–(number of mice showing HLE/total number of mice in each group) × 100 (35).

#### The chimney test

Acute adverse effects produced by the drugs alone and in combination were assessed using the chimney (motor performance) test (37). In this test, mice had to climb back up a vertical cylinder (30 cm in length and 3 cm in diameter) with a rough surface inside. The inability of the mouse to climb the cylinder in 60 s was considered as impaired locomotor capacity.

### Treatment plan

In experiment 1, the duration of HLE was assessed after the administration of different doses of methadone (0.1, 0.3, 1, and 3 mg/kg i.p.), and its vehicles after MES. The methadone doses were chosen according to a previous report (22).

In experiment 2, the duration of HLE was assessed after the administration of saline (5 ml/kg i.p.), valproate (300 mg/kg i.p.), and gabapentin (50 mg/kg i.p.) alone, after MES.

In experiment 3, the duration of HLE was determined after the administration of methadone + valproate sodium and methadone + gabapentin. Mice were first treated with saline or methadone before administration of valproate or gabapentin, and MES (22, 36).

Each animal was individually placed in the center of the plexiglass chamber during each trial.

### Biochemical measurements

At the end of the experiments, animals were sacrificed by decapitation under deep anesthesia. Next, the brain was immediately removed, weighed, and washed with 50 mM (pH 7.4) ice-cold PBS solution. Then, it was placed in 1/5 (w/v) PBS containing a protease inhibitor cocktail and homogenized for 30 s with 20-s intervals using an ultrasonic homogenizer (Hielscher, UP200H, Germany). The samples were centrifuged at 10,000 rpm for 10 min at 4°C (38), and supernatants were used to determine MDA, TNF levels, and protein contents.

### Measurement of brain tissue MDA and TNF-α

The tissue level of MDA and TNF-α contents was measured in the brain homogenates of mice after a single seizure with commercial kits (Karmania Pars Gene Company, Kerman, Iran), as previously described (29, 39). The concentrations of MDA and TNF-α were measured in brain homogenates that were prepared immediately after MES seizure. Protein concentration in brain homogenates was determined using Bradford's method (Karmania Pars Gene Company, Kerman, Iran). MDA concentration is expressed as nmol/mg protein tissue, and brain TNF-α levels are expressed as pg/mg protein.

### Statistical analysis

Results are expressed as HLE and percentage protection and mean ± SEM where applicable. The data were analyzed by one-way ANOVA and Tukey's *post-hoc* test (*p* ≤ 0.05) using GraphPad Prism 5.0. Results obtained with the chimney test are presented as the percentage of impaired mice in groups of 10 animals and were compared with Fisher's exact probability test. Statistical significance for biochemical assays was determined using two-way ANOVA for multigroup comparisons.

## Results

### Effect of methadone on MES-induced seizures in mice

Table 1 shows the significant effects of methadone (0.1, 0.3, 1, and 3 mg/kg i.p.), valproate sodium (300 mg/kg i.p.), and gabapentin (50 mg/kg i.p.) acute administration on MES-induced seizure (one-way ANOVA; F6, 63 = 0.8806, *p* < 0.0001). Tukey's multiple-comparisons *post-hoc* analysis showed that the duration of HLE was significantly (*p* < 0.001) reduced by administration of methadone (0.3 and 3 mg/kg i.p.) with protection percentage of 24 and 13.3, respectively. Treatment with sodium valproate (300 mg/kg) and gabapentin (50 mg/kg) alone decreased duration of HLE, and provided 53 and 45% protection against MES-induced seizure in mice.

**Table 1:** Effect of methadone, gabapentin, and sodium valproate, and their combination pretreatment on MES-induced seizures in mice.

**Treatment** **(mg/kg)**	**Duration (Mean ±SEM) of tonic hind limb extension (s)**	**Percent of protection**
Cont.	14.65 ± 0.3	0
GBP (50)	8.05 ± 0.57***	45
Val (300)	6.9 ± 0.61***	53
Met (0.1)	15.51 ± 0.31	0
Met (0.3)	11.13 ± 0.16***	24
Met (1)	15.7 ± 0.11	0
Met (3)	12.7 ± 0.5*	13.3
Met (0.1) + GBP (50)	18.73 ± 0.84^**##^	0
Met (0.3) + GBP (50)	13.64 ± 1.5^##^	6.8
Met (1) + GBP (50)	6.3 ± 0.12^**##^	56.9
Met (3) + GBP (50)	6.35 ± 0.19^**##^	56.9
Met (0.1) + Val (300)	13.86 ± 1.87^$$^	0
Met (0.3) + Val (300)	15.19 ± 2^$$^	24
Met (1) + Val (300)	18.67 ± 2.1^$$^	0
Met (3) + Val (300)	16.25 ± 3^$$^	13.3

Methadone (0.1, 0.3, 1, and 3 mg/kg i.p.), valproate sodium (300 mg/kg i.p.), and gabapentin (50 mg/kg i.p.) were injected before MES-induced seizure. Values are expressed as mean ± SEM; statistically significant test for comparison was done by ANOVA, followed by t-test (n = 10). Con, control; GBP, gabapentin; MES, maximal electroshock seizure; Met, methadone; Val, valproate sodium.

^*^p < 0.05, ^**^p < 0.01, ^***^p < 0.001 compared to the saline-treated control group. ##p < 0.001 compared to the MES group using one-way ANOVA followed by Tukey's test as a post-ANOVA test.

### Effect of methadone in combination with gabapentin or valproate on MES-induced seizures

Effects of methadone on anticonvulsant effects of gabapentin are shown in Table 1. Two-way ANOVA, including methadone (0.1, 0.3, 1, and 3 mg/kg) and gabapentin (50 mg/kg), revealed a significant effect (factor methadone, F4, 90 =19.36, *p* < 0.001; factor gabapentin; F1, 90 = 31.91, *p* < 0.001; factor methadone × gabapentin, F4, 90 = 19.3, *p* = 0.0001. *Post-hoc* analyses showed that methadone at doses of 0.1 and 0.3 mg/kg significantly (*p* < 0.001) decreased the effect of gabapentin on HLE duration, while at doses of 1 and 3 mg/kg it caused increased anticonvulsant effect of the gabapentin significantly (*p* < 0.001) with protection percentage of 56.9 and 56.9% (Table 1).

Moreover, the results (Table 1) showed a significant interaction between methadone (0.1, 0.3, 1, and 3 mg/kg) and valproate sodium (300 mg/kg) on MES (two-way ANOVA; factor methadone, F4, 90 = 4.98, *p* < 0.001; factor valproate sodium, F1, 90 = 5.69, *p* < 0.001, factor methadone × valproate sodium, F4, 90 = 6.2, *p* = 0.0002). Further analyses showed that methadone at all doses significantly (*p* < 0.05) reduced the duration of the convulsions and protection percentage of valproate sodium (300 mg/kg) against the MES-induced convulsions.

### Effects of different doses of methadone alone or in combination with valproate or gabapentin on motor coordination of mice after application of MES

The influence of the investigated compounds on motor coordination in mice is presented in Table 2. Our results showed that treatments of methadone both alone and combined with anticonvulsants had no impact on motor coordination in mice.

**Table 2:** Effect of methadone, gabapentin, and valproate sodium pretreatment *per se*, and combined therapy on motor coordination of mice in the chimney test.

**Treatments (mg/kg)**	**Time (s) ±SEM**
Con (Saline)	26.3 ± 1.73
GBP (50)	24 + 2.8
Val (300)	26 ± 2
Met (0.1)	22 ± 3
Met (0.3)	24 ± 1.9
Met (1)	25 ± 2.5
Met (3)	27 ± 3.2
Met (0.1) + GBP (50)	35 ± 5
Met (0.3) + GBP (50)	38 ± 5.2
Met (1) + GBP (50)	35 ± 6
Met (3) + GBP (50)	40 ± 4
Met (0.1) + Val (300)	29 ± 3
Met (0.3) + Val (300)	28 ± 3
Met (1) + Val (300)	25 ± 3
Met (3) + Val (300)	24 ± 3

Each value represents the mean ± SEM obtained from 10 mice. Statistical analysis: Student's t-test. The compounds and the vehicle were administered i.p. 60 min before the assay. Con, control; GBP, gabapentin; MES, maximal electroshock seizure; Met, methadone; Val, valproate sodium.

### Influence of pretreatment with different doses of methadone, gabapentin, and valproate *per se* on the brain MDA level in the mice after MES-induced seizure

**Figure 1:**
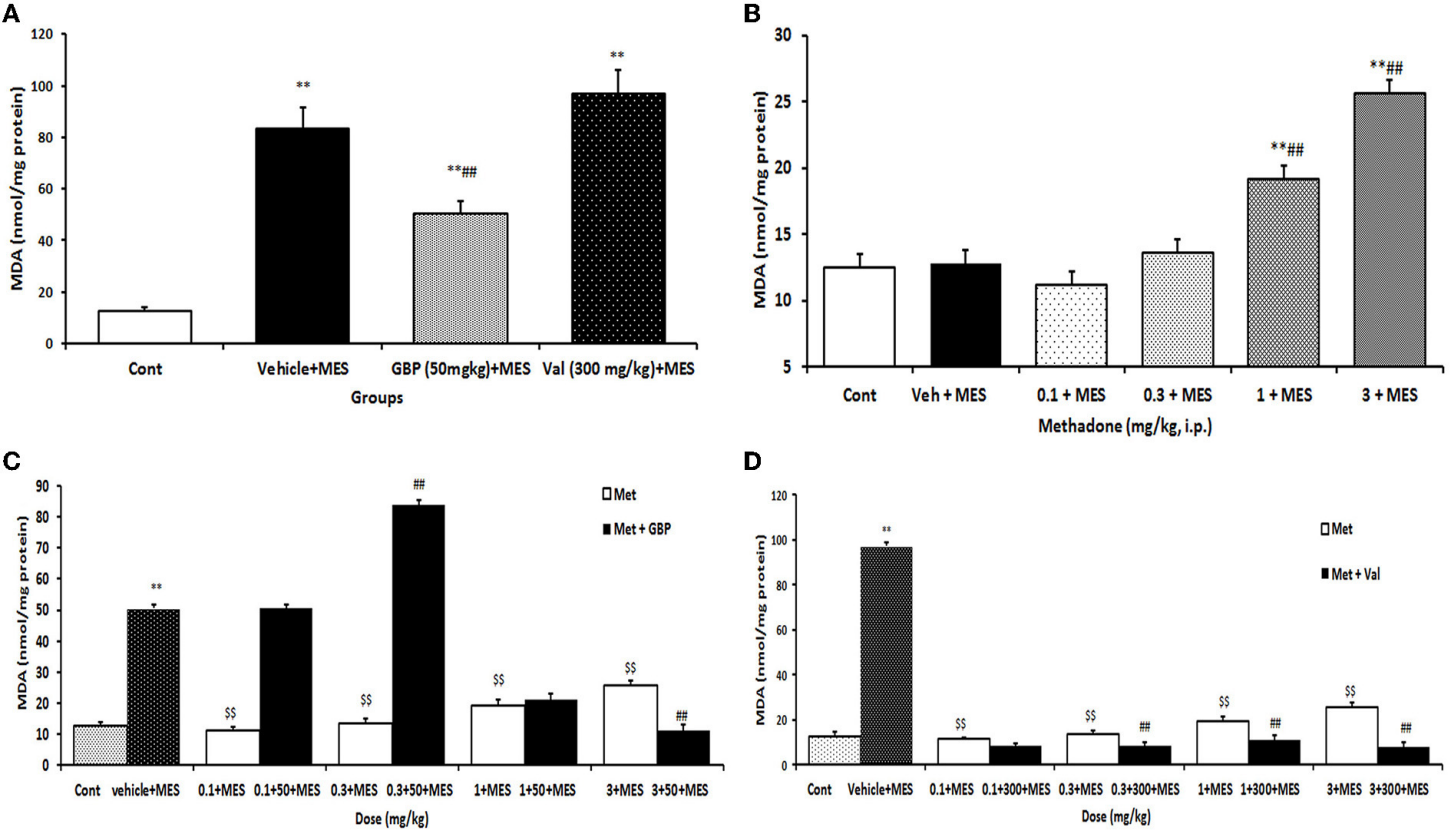
Changes of MDA (nmol/mg protein) in the brain of mice in different treatment groups: **(A)** changes of brain MDA level in the saline-treated control, 50 mg/kg i.p. gabapentin (GBP) and 300 mg/kg i.p. valproate (Val)-treated groups after MES-induced seizure; **(B)** changes of brain MDA level of mice after treatment with (0.1, 0.3, 1, and 3 mg/kg i.p.) Met and MES-induced seizure; **(C)** changes of brain MDA level treated with GBP (50 mg/kg i.p.) *per se* and different doses of Met plus GBP before MES; **(D)** changes of brain MDA level treated with valproate (Val) (300 mg/kg i.p.) *per se* and different doses of Met plus Val before MES. Data are expressed as mean ± SEM. *N* = 10. **p < 0.01compared with the normal group, $$*p* < 0.01 treatments vs. vehicle + MES, and ##*p* < 0.001 compared with the saline + MES group using one-way ANOVA followed by Tukey's test as a post-ANOVA test. Cont, control; GBP, gabapentin; Met, methadone; Val, valproate sodium; MES, maximal electroshock seizure.

There were significant differences in the mean brain MDA levels between different groups (*p* < 0.05). The results showed that brain MDA levels increased significantly (*p* < 0.001) after MES challenge compared to the control group. Pretreatment with gabapentin (50 mg/kg i.p.) or valproate sodium (300 mg/kg i.p.) *per se* significantly reduced brain level of MDA after MES administration (Figure 1A). Brain MDA content was significantly (*p* < 0.01) decreased following injection of all doses of methadone compared to vehicle-treated MES-induced seizure animals (Figure 1B).

### Influence of combined therapy with methadone and gabapentin or valproate on the brain MDA level in the mice after MES-induced seizures

Methadone at doses of 0.1 and 0.3 mg/kg i.p. decreased the reducing effect of gabapentin on MES-induced increase of MDA levels. But at higher doses it increased the suppressive effect of gabapentin on the increase in MDA level induced by MES in the brain (Figure 1C). Methadone at doses of 0.3, 1, and 3 mg/kg i.p. significantly increased the suppression of brain MDA levels produced by valproate after MES-induced seizures (Figure 1D).

### Influence of treatment with different doses of methadone, gabapentin, and valproate *per se* on the brain TNF-α level in MES-induced seizure in mice

**Figure 2:**
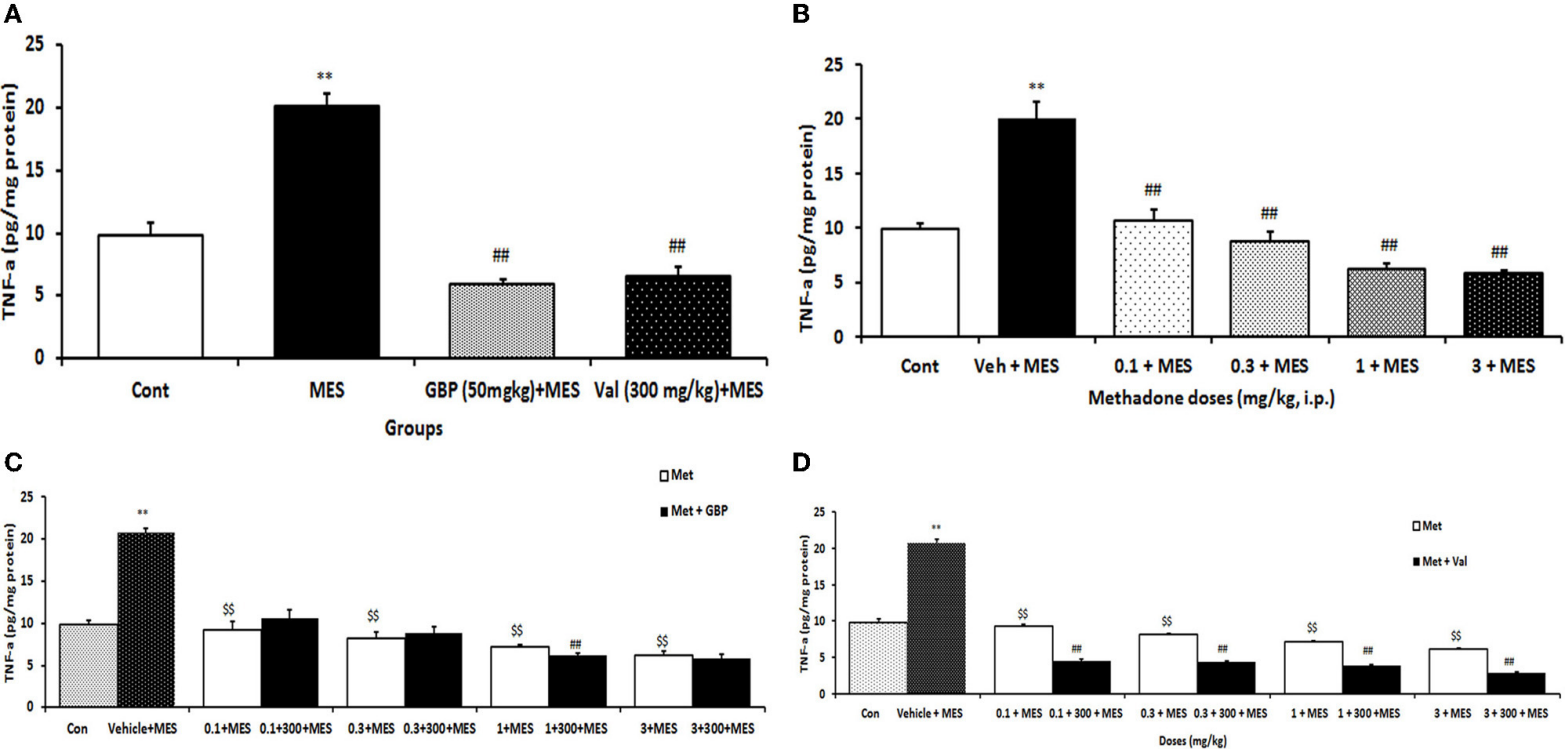
Changes of TNF-α (pg/mg protein) in the brain of mice in different treatment groups: **(A)** changes of brain TNF-α level in the saline-treated control, 50 mg/kg i.p. gabapentin (GBP) and 300 mg/kg i.p. valproate (Val)-treated groups after MES-induced seizure; **(B)** changes of brain TNF-α level of mice after treatment with (0.1, 0.3, 1, and 3 mg/k i.p.) Met and MES-induced seizure; **(C)** changes of brain TNF-α level treated with GBP (50 mg/kg i.p.) *per se* and different doses of Met plus GBP before MES; **(D)** changes of brain TNF-α level treated with valproate (Val) (300 mg/kg i.p.) *per se* and different doses of Met plus Val before MES. Data are expressed as mean ± SEM. N = 10. **p < 0.01 compared with the normal group, $$*p* < 0.01 treatments vs. vehicle + MES, and ##*p* < 0.001 compared with the saline + MES group using two-way ANOVA followed by Tukey's test as a post-ANOVA test. Cont, control; GBP, gabapentin; Met, methadone; Val, valproate sodium; MES, maximal electroshock seizure.

MES-induced seizures increased TNF-α levels significantly (*p* < 0.001) in mice brains compared to the control group. Treatment with both gabapentin (50 mg/kg i.p.) and valproate sodium (300 mg/kg i.p.) *per se* significantly (*p* < 0.01) prevented MES-induced TNF-α increase in the brain (Figure 2A). Treatment with all doses of methadone before MES-induced seizures suppressed the increase in the TNF-α levels in brain tissue (Figure 2B).

### Influence of combination therapy with methadone and gabapentin or valproate sodium on brain TNF-α level in mice following MES-induced seizure

Treatment with methadone significantly (*p* < 0.01) increased the suppressive effect of gabapentin on TNF-α level in the brain only at the dose 1 mg/kg (Figure 2C), while methadone at all doses increased the suppressive effect of valproate on the TNF-α level in the brain of mice receiving MES (Figure 2D).

## Discussion

Methadone is a drug used to treat morphine dependence that has been reported to show additional therapeutic action on inflammation and oxidative stress (40, 41). It is interesting that, in addition to opioid receptor agonist properties, methadone acts as a sodium and potassium channel blocker and NMDA receptor antagonist (24, 26). Both proconvulsant and anticonvulsant effects have been reported for NMDA receptor antagonists and potassium channels (42–44). Therefore, this study was designed to evaluate the potential impact of methadone alone or combined with two antiepileptic drugs on epilepsy and measure the amount of a lipid peroxidation product and cytokines in the brain.

In this study, we showed that pretreatment of mice with methadone resulted in a dual effect on the duration of the HLE in MES-induced seizures. Methadone at doses of 0.3 and 3 mg/kg significantly reduces this parameter compared to the control group. The percentage of seizure inhibition by methadone is 24 and 13.3% in 0.3 and 3 mg/kg dosage, respectively, compared to the control group. This effect is probably pharmacodynamic as there is no evidence that opioids affect the brain concentration of two antiepileptic drugs. This shows that methadone induced a U-shaped change in seizure suppression in comparison to the control group. These findings are consistent with a previous report that opioid agonists have a biphasic effect on seizure modulation (45). Furthermore, this study showed that the suppression of MES-induced seizures produced by gabapentin and valproate was affected by pretreatment with methadone. Therefore, we identified a new way to evaluate the relationship between opioids and anticonvulsants for either therapeutic purposes or drug interactions.

Similarly, a previous study has reported a dose-dependent effect of opioids in pentylenetetrazole (PTZ)-induced seizures, which supports the idea that methadone, as an opioid agonist, has a dual effect of inhibiting electrically induced seizures (46). Some publications are consistent with our results showing that opioid agonists have controversial dose-dependent effects on seizure activity in different animal seizure/epilepsy models (47–50). An earlier study has shown a reduction in the severity of PTZ-induced seizures after dynorphin treatment in mice, supporting our finding (51). Available experimental data indicated that opioid agonists, regardless of their action on the seizure threshold, showed potential for changing the effects of antiepileptic drugs (52).

In contrast, many experiments have shown that the interaction of opioid agonists with the agents has shown an anticonvulsant effect (53, 54). Therefore, the association between opioid receptor agonists and anticonvulsant drugs is balanced between increasing and decreasing effects on the seizure threshold. However, only a few studies examined the interaction between opioid agonists, anticonvulsant drugs, and possible molecular mechanisms of the disease. Therefore, we evaluated the effect of methadone treatment on the efficacy of two anticonvulsant drugs and the possible mechanisms involved in these conditions.

The induction of seizure by MES represents an experimental model of tonic–clonic seizures with similarities to human seizures (55, 56). The model has also shown high sensitivity to compounds affecting opioidergic mechanisms (57). Prolonged tonic phase of the seizure has been found to affect antioxidant enzyme activity and increase lipid peroxidation in the brain (58, 59).

In this study, methadone administration resulted in (1) reduced duration of HLE; (2) increased percentage of protection against electroshock-induced seizure; (3) different effects on the efficacy of gabapentin and valproate; (4) attenuation of the brain MDA and TNF-α levels; and (5) no adverse effects on motor coordination.

The findings of this study are in concordance with an earlier study in which combined treatment with a plant extract with anti-inflammatory and antioxidant activity and valproate suppressed the development of seizures more effectively in patients with epilepsy who were refractory to standard antiepileptic treatment (60). Similarly, it was reported that a plant extract containing antioxidants enhanced the antiseizure effect of carbamazepine in an experimental model of acute seizures (61).

To explain the observed alteration in the antiseizure effects of gabapentin and valproate by methadone, one should consider the molecular mechanisms of action of these drugs on brain neurotransmitters and neuronal ion channels under modulatory control of MDA and TNF-α. Many studies have shown that an increase in lipid peroxidation levels in the brain has a pivotal role in epilepsy that in turn ignites the neuroinflammation cascades in the brain (62, 63). Scientific evidence indicates that success in the treatment of seizure is associated with the suppression of neuroinflammation, excitotoxicity, and enhancement of inhibitory neurotransmission in the brain (64). Also, a reciprocal relationship exists between inhibitory neurotransmission, reactive oxygen species, and TNF-α levels in the brain during the seizure (65–67). In this study, the MES group exhibited significant induction in lipid peroxidation and TNF-α production in the mice brains. This is in line with the previously described association between biosynthesis of cytokines and acute seizures in rodents (68). Methadone administration was able to impede the elevation of MDA and TNF-α in the MES-induced seizure in mice brains, suggesting the probable antioxidative and anti-inflammatory effect of methadone that may contribute to its protective action toward seizure and antiepileptic medication. Previous investigations showed a correlation between plasma levels of cytokine and methadone maintenance therapy outcomes in patients with opioid use disorder (69).

Moreover, methadone has demonstrated anti-inflammatory activity by activating opioid receptors (16). Several publications have shown a relationship between increased lipid peroxidation and cytokines levels in the brain and change in the potency of anticonvulsant drugs (70, 71). Oxidative stress and inflammation are interrelated mechanisms involved in the regulation of neural activity, and increasing their levels in the brain may contribute to hyperexcitability and epilepsy (72). In addition to its direct effect on GABA receptors, gabapentin has been shown to have anti-inflammatory effects that may contribute to its neuroprotective effect against seizure severity (68, 73). Previous investigations using a neuropathic pain model showed a synergistic effect between anti-inflammatory agents and gabapentin (74).

However, valproate exerts its effects by increasing the concentrations of GABA *via* inhibiting its metabolism and increasing its synthesis (75). Therefore, there is still a significant and positive association between an increase in the oxidative stress markers and a decrease in the antioxidant levels with the number of seizures in valproate monotherapy (65).

In this study, we observed increased MDA and TNF-α levels in the brains of mice after MES, suggesting their role in epileptogenesis, which is in line with the previous studies where seizure induced excitotoxicity and increase in oxidative stress and cytokine levels in the brain (76). Our results also demonstrated that administration of methadone increased brain tissue content of MDA while decreasing TNF-α levels, suggesting their role in the development of MES-induced seizures. Furthermore, the administration of methadone and the anticonvulsant drugs enhanced their suppressive effect on the levels of MDA and TNF-α in the brains of mice at effective anticonvulsant doses. Another study revealed the improvement of mortality rate in kainic acid-induced epilepsy by inhibiting the inflammation and oxidant stress (77). Furthermore, recent evidence has documented that suppression of oxidative stress/cytokine production contributes to the antiseizure potency of anticonvulsant drugs (78).

MDA, as the by-product of the peroxidation of polyunsaturated fatty acids, is toxic to neurons and plays a crucial role in the induction of neuroinflammation. An increase in its brain levels leads to the progression of epilepsy through destabilization of the lipid membrane (79). There is a positive correlation between plasma MDA level and seizure severity in patients with epilepsy (80, 81). In the pilocarpine-induced seizure model, administration of antioxidants could protect the neuron's lipid peroxidation and nitrite formation-induced damages (82, 83). This is in line with a previous study that reported that MES-induced seizures increased oxidative stress, neurotoxicity, and cognitive impairment (84). The administration of methadone *per se* and in combination with antidepressants was able to suppress lipid peroxidation and inflammation in the brains of mice receiving MES, suggesting the potential anti-inflammatory and antioxidant activity of methadone. Therefore, methadone at specific doses is regarded as an anticonvulsant adjuvant and protects the brain from seizure damage.

TNF-α is an endogenous cytokine whose rise in the brains of rodents is caused by seizure activity and clinical subjects after seizure activity (85). In the central nervous system, TNF-α as a pro-inflammatory cytokine has a regulatory action on neuronal excitability and myelin stability under physiological conditions (86). TNF-α is both produced by neurons in physiological conditions (32) and overproduced by overactive microglia under pathological conditions (87). Recent studies have shown paradoxical effects of TNF-α on seizure (88, 89). This finding may partly explain why methadone administration alone and in adjunctive therapy with an anticonvulsant regimen failed to completely suppress seizure responses. Indeed, other factors, such as the regulation of GABA release, may mediate these effects (90, 91). Today, it is known that the narrow and different therapeutic dosage range of antiepileptic compounds can be caused by the differences in the mechanisms of epilepsy induction in acute and chronic models or the different methods of drug administration (92, 93).

The data in this study report the controversial effect of methadone *per se* and in combination therapy gabapentin and valproate effects on MES-induced seizures in mice. Accordingly, this study showed that administration of methadone with gabapentin and valproate in mice with MES-induced seizure affected brain MDA and TNF-α levels, which might be possible mechanisms of methadone effects on the modulation of anticonvulsant drugs. Further studies are required to establish the exact mechanism of action of the opioid agonists on the modulation of anticonvulsant drugs and warrant further investigations.

## Conclusion and perspectives

The data in this study are the first report of biphasic anticonvulsant and proconvulsant effects of methadone on MES-induced seizures. In addition, the data show that methadone interacts with the anticonvulsive effects of valproate sodium and gabapentin, which were free from adverse effects on motor coordination. Specifically, the mix of methadone as an opioid receptor agonist and gabapentin and valproate as anticonvulsants display increased and decreased potency, respectively, in mice with MES-induced seizure. The diverse effects of methadone pretreatment shown here make it important for its potential application in human translation studies and aimed at preventing inappropriate drug therapeutic interactions in the treatment of epilepsy. Moreover, brain levels of MDA and TNF-α are shown to serve as viable candidates for drug interactions efficacy monitoring, hence facilitating the quest for antiepileptic drug therapies. The mechanisms responsible for these effects are unclear from this study but seem to involve changes in brain MDA and TNF-α levels.

## Data availability statement

The raw data supporting the conclusions of this article will be made available by the authors, without undue reservation.

## Ethics statement

The animal study was reviewed and approved by Ethical Committee of Shahed University with the code number: IR.SHAHED.REC.1400.072.

## Author contributions

AM performed experiments, assessed, and prepared the data. MH co-performed animal experiments, assessed and analyzed data, designed and coordinated the study, interpreted data and wrote the article. All authors contributed to the article and approved the submitted version.

## Conflict of interest

The authors declare that the research was conducted in the absence of any commercial or financial relationships that could be construed as a potential conflict of interest.

## Publisher's note

All claims expressed in this article are solely those of the authors and do not necessarily represent those of their affiliated organizations, or those of the publisher, the editors and the reviewers. Any product that may be evaluated in this article, or claim that may be made by its manufacturer, is not guaranteed or endorsed by the publisher.
